# Identification of Differentially Expressed Genes and miRNAs for Ulcerative Colitis Using Bioinformatics Analysis

**DOI:** 10.3389/fgene.2022.914384

**Published:** 2022-06-02

**Authors:** Weitao Hu, Taiyong Fang, Xiaoqing Chen

**Affiliations:** ^1^ Department of Rheumatology, The Second Affiliated Hospital of Fujian Medical University, Quanzhou, China; ^2^ Department of Gastroenterology, The Second Affiliated Hospital of Fujian Medical University, Quanzhou, China

**Keywords:** ulcerative colitis, bioinformatics analysis, differentially expressed genes, microarray, protein-protein interaction, MicroRNAs

## Abstract

**Introduction:** Ulcerative colitis (UC) is a chronic inflammatory disease of the intestine whose cause and underlying mechanisms are not fully understood. The aim of this study was to use bioinformatics analysis to identify differentially expressed genes (DEGs) with diagnostic and therapeutic potential in UC.

**Materials and methods:** Three UC datasets (GSE179285, GSE75214, GSE48958) were downloaded from the Gene Expression Omnibus (GEO) database. DEGs between normal and UC tissues were identified using the GEO2R online tool. The Gene Ontology (GO) and Kyoto Encyclopedia of Genes and Genomes (KEGG) pathway enrichment analyses of the DEGs were performed using Metascape. Protein-protein interaction network (PPI) analysis and visualization using STRING and Cytoscape. Finally, the miRNA gene regulatory network was constructed by Cytoscape to predict potential microRNAs (miRNAs) associated with DEGs.

**Results:** A total of 446 DEGs were identified, consisting of 309 upregulated genes and 137 downregulated genes. The enriched functions and pathways of the DEGs include extracellular matrix, regulation of cell adhesion, inflammatory response, response to cytokine, monocarboxylic acid metabolic process, response to toxic substance. The analysis of KEGG pathway indicates that the DEGs were significantly enriched in Complement and coagulation cascades, Amoebiasis, TNF signaling pathway, bile secretion, and Mineral absorption. Combining the results of the PPI network and CytoHubba, 9 hub genes including CXCL8, ICAM1, CXCR4, CD44, IL1B, MMP9, SPP1, TIMP1, and HIF1A were selected. Based on the DEG-miRNAs network construction, 7 miRNAs including miR-335-5p, mir-204-5p, miR-93-5p, miR106a-5p, miR-21-5p, miR-146a-5p, and miR-155-5p were identified as potential critical miRNAs.

**Conclusion:** In summary, we identified DEGs that may be involved in the progression or occurrence of UC. A total of 446 DEGs,9 hub genes and 7 miRNAs were identified, which may be considered as biomarkers of UC. Further studies, however, are needed to elucidate the biological functions of these genes in UC.

## Introduction

UC is a group of chronic inflammatory diseases of the intestine whose etiology is not yet fully understood. UC begins in the rectum and usually extends proximally in a continuous manner through part or the entire colon (Danese and Fiocchi, 2011; Ordás et al., 2012). The typical clinical manifestations of UC are bloody diarrhea, abdominal pain, fecal urgency, and tenesmus. In some patients, extraintestinal manifestations may precede the onset of gastrointestinal symptoms (Feuerstein et al., 2019). Globally, UC is more common than Crohn’s disease (Ng et al., 2017), and a variety of drugs are available for its treatment (Ungaro et al., 2017). However, up to 15% of cases do not respond to medication or have chronic colitis secondary to dysplasia and require surgical treatment (Dignass et al., 2012). For this reason, understanding the precise molecular mechanisms underlying UC is vital for developing therapeutic approaches.

Bioinformatics is an emerging discipline that is considered as one of the promising and critical approaches for early diagnosis, effective treatment and prediction of clinical challenges in the prognosis of cancer patients (Wang and Liotta, 2011). This new strategy has been widely used in the study of various cancers (Li et al., 2017; Yan et al., 2018; Tsai and Gamblin, 2019), and has been instrumental in the identification of novel biomarkers in several non-tumor diseases (Chen et al., 2018; Cakmak and Demir, 2020; Xie et al., 2020).

Bioinformatics and microrray technology are widely used in screening for genetic alterations at the genomic level, and to search DEGs and functional pathways participating in the development and progression of inflammatory bowel disease (IBD). However, independent microarray analysis makes it difficult to get reliable results because of its high false-positivity rate. Thus, in the present study, to identify DEGs between normal and UC colonic mucosa tissues, 3 mRNA microarray datasets were downloaded from Gene Expression Omnibus (GEO). Gene Ontology (GO), Kyoto Encyclopedia of Genes and Genomes (KEGG) pathway enrichment analysis and protein-protein interaction (PPI) network analyses were then conducted to identify molecular mechanisms underlying the development and progression of UC. Finally, the miRNA gene regulatory network was constructed using Cytoscape to predict potential microRNAs (miRNAs) associated with DEGs. In conclusion, a total of 446 DEGs, 9 hub genes and 7 potential miRNAs were identified as candidate biomarkers for UC.

## Materials and Methods

### Microarray Data

GEO (http://www.ncbi.nlm.nih.gov/geo) (Edgar et al., 2002) is a public functional genomics data repository of high throughput gene expression data, chips and microarrays. The GSE75214 (Vancamelbeke et al., 2017) and GSE48958 (Van der Goten et al., 2014) datasets generated using the Affymetrix GPL6244 platform (Affymetrix Human Genome 1.0 ST Array), and GSE179285 (Keir et al., 2021) generated on the GPL6480 platform (Agilent-014850 Whole Human Genome Microarray 4 × 44K G4112F) were downloaded from GEO. Annotated information from the platform was used to convert the probes to the corresponding gene symbols. The GSE179285 dataset contained 23 UC inflamed colonic mucosa tissue samples and 23 control samples; the GSE75214 dataset contained 74 biopsies from inflamed colonic mucosa of active UC patients and 11 healthy control samples; and the GSE48958 dataset contained 7 colonic mucosal biopsies from UC patients with active disease and 8 colonic mucosal biopsies from controls.

### Identification of DEGs

Identification of DEGs between UC and normal samples was performed using GEO2R (http://www.ncbi.nlm.nih.gov/geo/geo2r). GEO2R is an online interactive tool that allows users to identify DEGs for different experimental conditions by comparing two or more datasets in the GEO series. Adjusted p-values (adj. P) and Benjamini and Hochberg’s false discovery rates were applied to provide a balance between discovering statistically significant genes and limiting false positives. Probe sets without corresponding gene symbols or genes with more than one probe set were deleted or normalized, respectively. Log FC (fold change) >1 and adj. *p*-value <0.01 were considered statistically significant.

### KEGG and GO Enrichment Analyses of DEGs

Metascape (https://metascape.org/gp/index.html#/main/step1) (Yingyao Zhou et al., 2019) is an analytical website that integrates functional enrichment, interactomic analysis, genetic annotation, and membership search, utilizing more than 40 individual knowledge bases in a comprehensive portal. KEGG is a database resource for elucidating high-level functions and effects of biological systems (Kanehisa, 2002; Kanehisa et al., 2017). GO is a major bioinformatics initiative for high-quality functional gene annotation based on biological processes (BP), molecular functions (MF) and cellular components (CC) (Pomaznoy et al., 2018). Metascape was used to predict the functions of DEGs, with the screening conditions set as Min overlap = 3 and Min Enrichment = 1.5. *p* < 0.01 was considered statistically significant.

### PPI Network Construction and Module Analysis

The PPI network was constructed using the Search Tool for the Retrieval of Interacting Genes (STRING; http://string-db.org) (version 11.5) (Franceschini et al., 2013) online database, and the parameters were set as follows: meaning of network edges: confidence level; minimum required interaction score: medium confidence (0.400); active interaction sources: Select Textmining, Experiments, Databases, Co-expression, Neighborhood, Gene Fusion and Co-occurrence. Identification of functional interactions between proteins may provide insights into the mechanisms underlying the development of diseases. Cytoscape (version 3.8.2) is an open-source bioinformatics software platform for visualizing molecular interaction networks (Smoot et al., 2011). Molecular Complex Detection (MCODE) (version 2.0) is a plug-in in Cytoscape used to identify densely connected regions by clustering a given network based on the topology (Bandettini et al., 2012). Using Cytoscape to map the PPI network, the MCODE was used to identify the most significant modules in the PPI network. The following selection criteria were used: MCODE scores >5, degree cut-off = 2, node score cut-off = 0.2, Max depth = 100 and k-score = 2.

### Selection and Analysis of Hub Genes

The top 9 genes were obtained using MCC algorithm with Cytoscape’s plug-in cytoHubba. Metascape was used to predict the functions of hub genes, with the screening conditions set as Min overlap = 3 and Min Enrichment = 1.5. *p* < 0.01 was considered statistically significant.

### MiRNAs Associated With Hub Genes

The top 9 hub genes were mapped to the respective miRNAs with NetworkAnalyst 3.0 (Guangyan Zhou et al., 2019) (https://www.networkanalyst.ca/), an online platform for visualization that helps to identify miRNA-gene interactions in Gene Regulatory Networks. For each hub gene, miRNAs were identified as having a degree cutoff = 1.0. Lastly, a mapping of these hub genes and miRNAs was performed by Cytoscape 3.8.2.

## Results

### Identification of DEGs in UC

**FIGURE 1:**
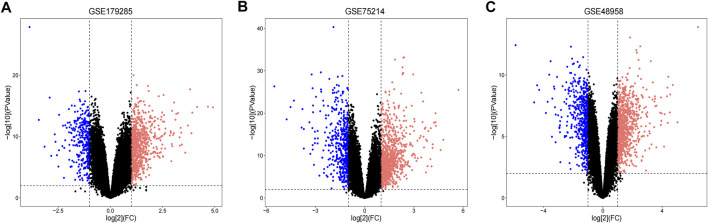
Volcano plots indicating differentially expressed genes (DEGs) among the control and UC groups. **(A–C)** DEGs of the GSE179285, GSE75214 and GSE48958 datasets are shown, separately. red data points represent up-regulated genes and blue ones represent down-regulated genes. Genes without any significant differences are in black.

Three datasets (GSE179285, GSE75214 and GSE48958) containing gene expression profiles of healthy and UC inflamed/active colonic mucosa tissue samples were retrieved from the GEO database. Details for the three datasets are presented in Table 1. After standardization of the microarray results, DEGs (894 in GSE179285, 1236 in GSE75214 and 1244 in GSE48958) were identified. The DEGs in the GSE179285, GSE75214 and GSE48958 datasets included 634 upregulated and 260 downregulated genes, 790 upregulated and 446 downregulated genes and 839 upregulated and 405 downregulated genes, respectively. All DEGs were identified by comparing gene expression profiles between healthy controls and UC inflamed/active samples. The gene expression profiles of DEGs in the three datasets containing 2 sets of sample data are shown in Figure 1.

**TABLE 1:** Details for GEO UC data.

Reference	GEO	Platform	Control	UC
Van der Goten J(2014)	GSE48958	GPL6244	8	7
Vancamelbeke M(2017)	GSE75214		11	74
Keir ME (2021)	GSE179285	GPL6480	23	23

**FIGURE 2:**
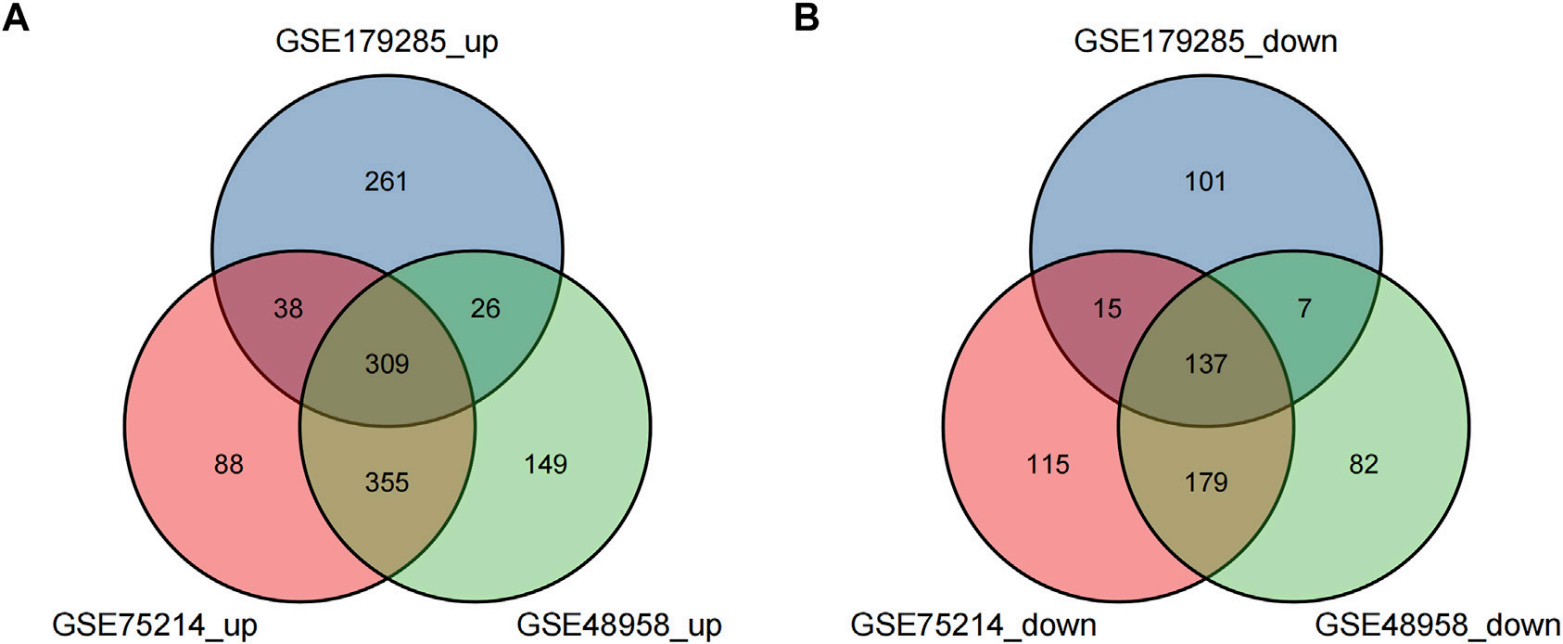
Venn diagrams showing the differentially expressed genes (DEGs) that overlapped among the 3 datasets retrieved from Gene Expression Omnibus (GEO). **(A,B)** indicate the overlap of up-regulated and down-regulated genes in the GSE179285, GSE75214, and GSE48958 datasets, separately.

These genes were screened further and Venn diagrams were mapped to represent these genes. As shown in Figure 2, 446 DEGs were found to be significantly differentially expression in 3 groups, of which 309 genes were upregulated and 137 genes were downregulated (Table 2).

**TABLE 2:** Screening DEGs in UC patients by integrated microarray.

DEGs	Gene terms
Upregulated	ABCA12 ACKR4 ADAM19 ADAMTS1 ADAMTS9 ADGRF1 ADGRG6 ADM AGPAT4 AGT ANGPT2 ANGPTL2 ANGPTL4 ANO6 ANXA1 APOL1 AQP9 AREG ARFGAP3 ARHGDIB ARID5A ARNTL2 ASPHD2 BACE2 BASP1 BCL2A1 BIRC3 BST2 C1R C1S C2 C3 C4BPA C4BPB C8orf4 CARD6 CASP1 CASP4 CASP5 CAV1 CCDC3 CCL20 CD34 CD38 ***CD44*** CD55 CD79A CDH11 CDH3 CEBPB CEMIP CFB CFI CHI3L1 CHI3L2 CHST15 CLU COL12A1 COL15A1 COL1A1 COL4A1 COL4A2 COL6A3 CREB3L2 CTSE CTSH CTSK CXCL1 CXCL11 CXCL2 CXCL3 ***CXCL8*** CXCL9 ***CXCR4*** CXorf36 CYP4X1 CYR61 CYTIP DAPP1 DCN DERL3 DMBT1 DRAM1 DUOX2 DUOXA2 DYSF EGR1 ELK3 ELL2 EMB ERG F2RL2 FADS1 FADS2 FAM20A FAM65B FAP FBN1 FCGR3A FCN3 FCRL5 FKBP11 FLI1 FOS FPR1 FSTL1 FUT8 FXYD5 FYN G0S2 GABRP GBP4 GBP5 GEM GJA1 GLCCI1 GMFG GNG11 GPR183 GPX7 GPX8 GREM1 GUCY1A3 GZMK HCAR3 HEG1 ***HIF1A*** HLA-DMA ***ICAM1*** ICAM2 ICOS IDO1 IFI16 IFI6 IFITM2 IFITM3 IGFBP5 IGFBP7 IGHV3-69-1 IGKC IKBIP ***IL1B*** IL1RN IL2RA IL33 IL7R INPP5D IRAK3 ITGAX ITGB2 JAK3 KDELR3 KLK10 KYNU LAMC1 LAMP3 LAX1 LCN2LCP1 LEF1LHFP LILRB3 LIPG LOXL2 LPCAT1 LRP8 LUM LYN MCAM ME1 MEDAG MGP MMP1 MMP10 MMP12 MMP3 MMP7 ***MMP9*** MNDA MSN MUC1 MXRA5 MYEOV MZB1 NCF2 NFKBIZ NNMT NOS2 NR4A1 OLFM4 ORAI2 PARP8 PCDH17 PCOLCE PCSK1 PDE4B PDPN PDZK1IP1 PEA15 PECAM1 PHLDA1 PI3 PIK3R3 PIM2 PITPNC1 PLA1A PLA2G2A PLAU PLEKHS1 PLS3 PLXND1 PMP22 PODXL POU2AF1 PROK2 PSMB9 PTAFR PTPRC PTRF PXDN RAB31 RAC2 RBMS1 RBPMS REG1A REG1B REG4 RFTN1 RGCC RGS1 RGS2 RGS5 ROBO1 RPS6KA2 S100A11 S100A8 S100A9 S100P S1PR1 SAA1 SAA2 SAMD9L SCD SDR16C5 SELE SELP SERPINA3 SERPINB3 SERPINB4 SERPINB5 SERPINB7 SLA SLC28A3 SLC5A1 SLC6A14 SLC6A20 SLC6A6 SLCO1B3 SLFN11 SNX10 SOCS1 SOCS3 SPARC SPARCL1 SPNS2 ***SPP1*** SRD5A3 SRGN SRPX2 ST3GAL1 STOM SULF1 TAP1 TBXAS1 TCF4 TCN1 TEK TFF1 TFPI TFPI2 TGFBI TGM2 THBS2 THEMIS2 THY1 ***TIMP1*** TIMP3 TM4SF1 TMEM45A TMEM92 TMTC1 TNC TNIP3 TRIB2 TRIM22 TRIM29 TSPAN11 UBD UNC13D VNN1 VNN2 VSIG1 VWF WARS WISP1 WNT5A XBP1 ZBP1 ZC3H12A
Downregulated	A1CF ABCB1 ABCG2 ACADS ACAT1 ACOX1 ACSF2 ACVR1C ADH1A ADH1C AHCYL2 AIFM3 AKR1B10 ANO5 ANPEP APOBEC3B AQP8 ARHGAP44 B4GALNT2 BEST2 BRINP3 C2orf88 CA1 CA12 CA2 CAPN13 CCNJL CDHR1 CDKN2B CHP2 CIPC CKB CLCN2 CLDN8 CLYBL CMBL CNTN3 CWH43 CYP2B6 CYP4F12 CYP4F2 DDC DHRS11 EDN3 ENTPD5 EPHX2 ETFDH EXPH5 FGFR2 FGFR3 GBA3 GRAMD1C GUCA2A GUCA2B GXYLT2 HEPACAM2 HMGCS2 HPGD HSD17B11 HSD17B2 HSD3B2 IGSF9 KCNK5 LEAP2 LRRN2 MCOLN2 MEP1B METTL7A MS4A12 MT1E MT1F MT1G MT1JP MT1M MTMR11 NAAA NAT8B NR1H4 NXPE1 NXPE2 P2RY1 PADI2 PAQR5 PBLD PCK1 PDE6A PDK2 PHLPP2 PHYH PIGZ PKIB PLA2G12B PPARG PPARGC1A PPP2R3A PRDX6 PRR15 PTGDR PTPRR PXMP2 RHOU RMDN2 RUNDC3B SATB2 SCIN SCUBE2 SELENBP1 SETD9 SGK2 SLC16A1 SLC16A9 SLC17A4 SLC22A5 SLC23A3 SLC26A2 SLC30A10 SLC35G1 SLC38A4 SLC39A5 SLC3A1 SLC4A4 SLC51A SLC51B SUGCT SULT1A2 TDP2 TRIM36 TRPM6 TSPAN7 TUBAL3 UGDH UGT2A3 VIPR1 VLDLR WDR78 WSCD1 ZDHHC23

### KEGG and GO Enrichment Analyses of DEGs

**FIGURE 3:**
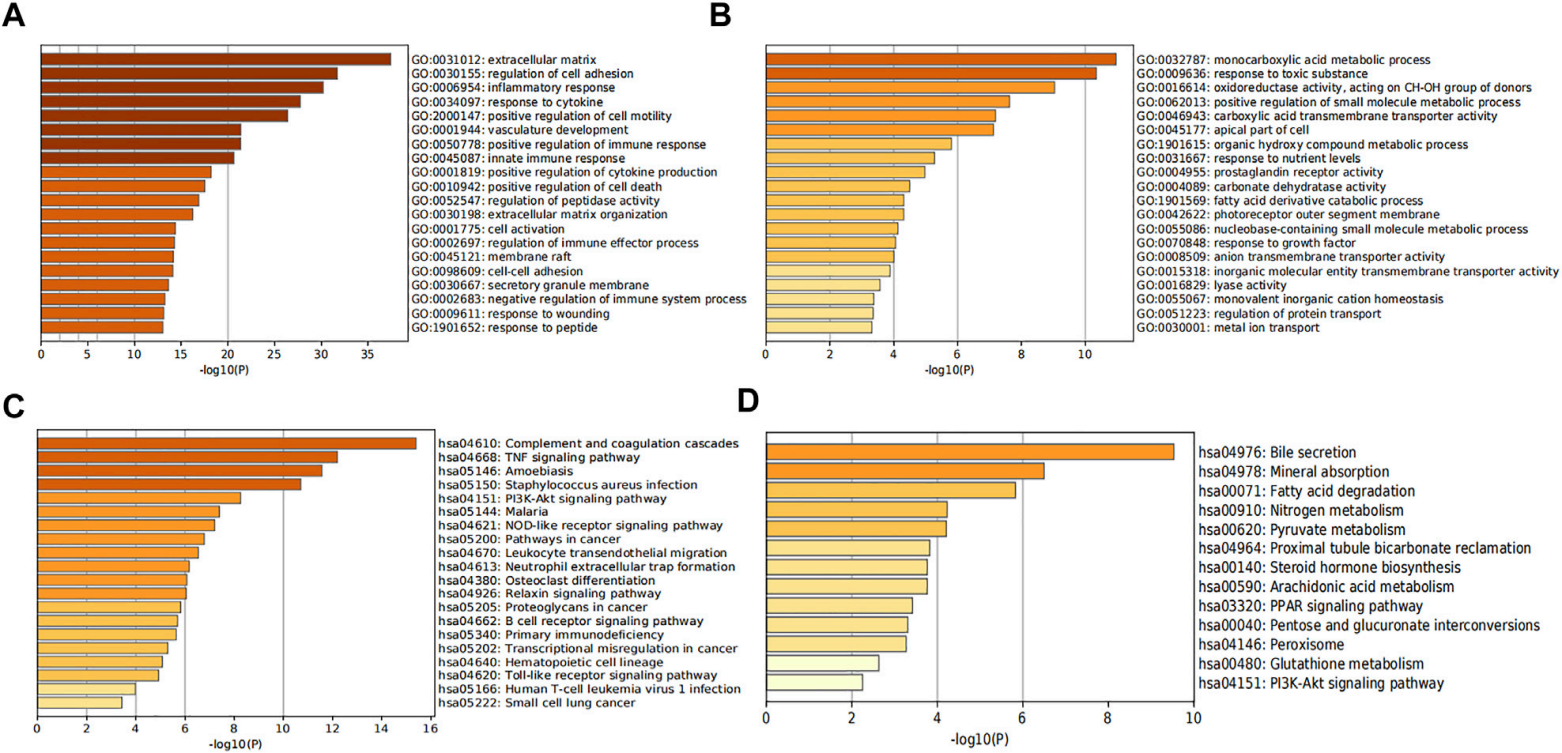
Analysis of functional enrichment in DEGs. The bar graphs show the top 20 results of the up-regulated gene **(A,C)** and down-regulated gene **(B,D)** enrichment analysis. *p*-value is displayed in color.

To predict the biological functions of the DEGs, we performed functional enrichment analysis of up-regulated and downregulated genes. Results of GO analysis showed that the up-regulated genes were mainly enriched in extracellular matrix, regulation of cell adhesion, inflammatory response and response to cytokines (Figure 3A, Supplementary Table S1), while the down-regulated genes were significantly enriched in monocarboxylic acid metabolic process and response to toxic substances (Figure 3B, Supplementary Table S2). KEGG pathway analysis indicated that the up-regulated genes were significantly enriched in Complement and coagulation cascades, Amoebiasis and TNF signaling pathway (Figure 3C, Supplementary Table S3), while the down-regulated genes were mainly enriched in bile secretion and Mineral absorption (Figure 3D, Supplementary Table S4).

### PPI Network Construction, Module Analysis and Hub Genes Identification

**FIGURE 4:**
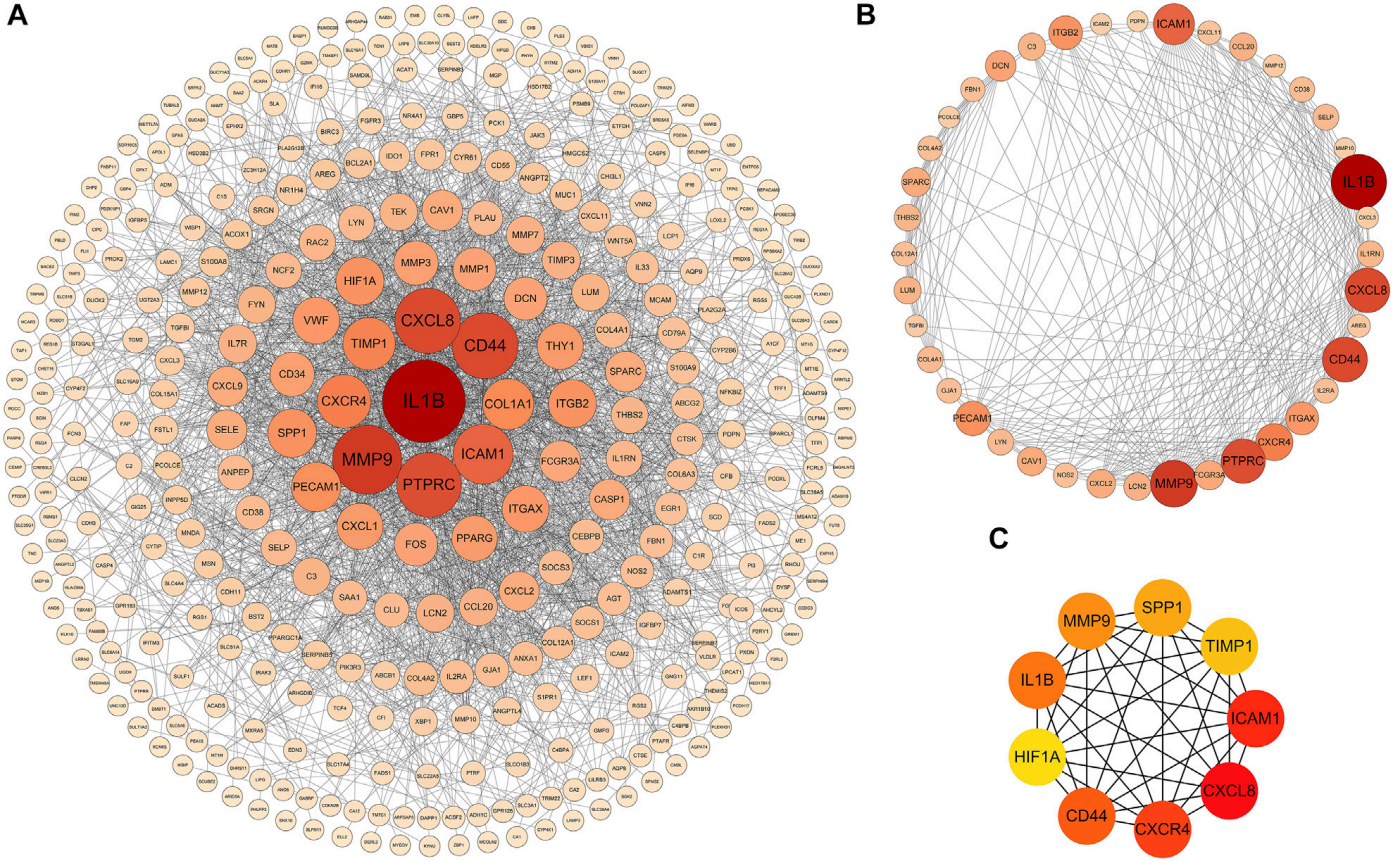
PPI networks of 309 up-regulated genes and 137 down-regulated genes by Cytoscape. The network consists of 446 nodes and 2163 edges. Two edges between nodes represent the interactions between genes. Each gene corresponding to the node is sized and colored according to the degree of interaction. The color grade indicates the change in the degree of each gene from high (orange-red) to low (faint yellow). The nearer the red node, the higher the connection between the Two nodes **(A)**. The densest connected region in the PPI network (40 nodes, 275 edges) was identified using MCODE **(B)**. Using the MCC algorithm in cytoHubba, 9 hub genes were identified in the densest connected regions. The scores are shown in red color. A darker color means a higher score **(C)**.

PPI analysis of the DEGs was based on the STRING database and the results were visualized using Cytoscape (Figure 4A). Using MCODE, a plug-in in Cytoscape, we identified the most densely connected regions (40 nodes, 275 edges) in the PPI network (Figure 4B). The top 9 genes, including CXCL8, ICAM1, CXCR4, CD44, IL1B, MMP9, SPP1, TIMP1, and HIF1A, were obtained using MCC algorithm with Cytoscape’s plug-in cytoHubba (Figure 4C). The results showed that CXCL8 (Chemokine (C-X-C motif) Ligand 8, score 3.24E+11), ICAM1 (Intercellular Adhesion Molecule 1, score 3.24E+11), CXCR4 (Chemokine (C-X-C motif) Receptor 4, score 3.24E+11), CD44 (CD44 Molecule, score 3.24E+11) and IL1B (Interleukin 1 Beta, score3.24E+11) were the most significant genes, followed by MMP9 (Matrix Metallopeptidase 9, score 3.23E+11), SPP1 (Secreted Phosphoprotein 1, score 3.20E+11), TIMP1 (TIMP Metallopeptidase Inhibitor 1, score 3.14E+11) and HIF1A (Hypoxia Inducible Factor 1 Subunit Alpha, score 3.13E+11).

### Analysis of Hub Genes

**FIGURE 5:**
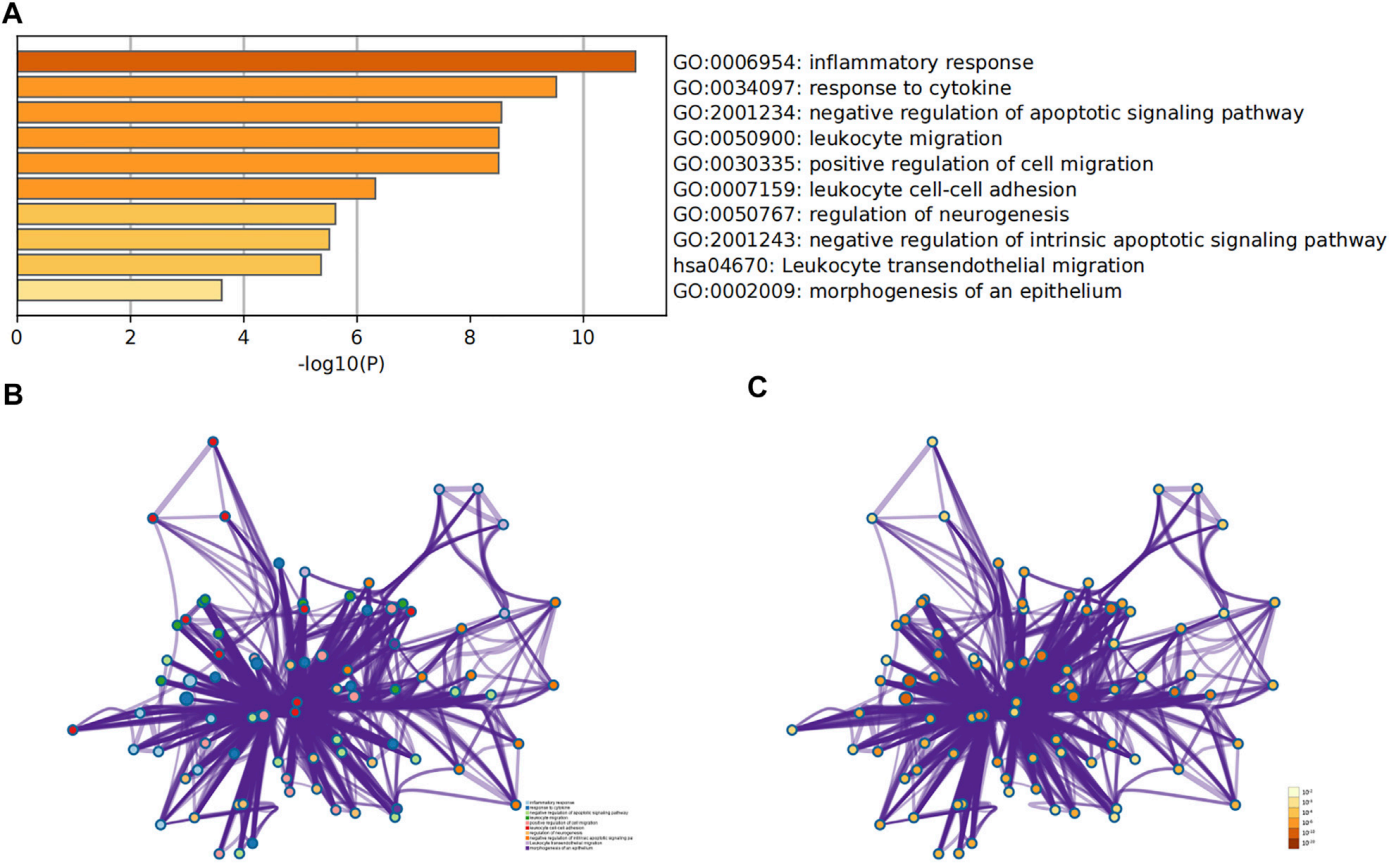
Analysis of functional enrichment for hub genes. **(A)** Bar graph of GO analyses for hub genes. P-value is shown in color. Enrichment term network of hub genes; colors indicate the same cluster ID **(B)**, and P-value **(C)**.

The hub gene symbols, abbreviations and functions are shown in Table 3. Functional enrichment analysis showed that the 9 hub genes were mainly concentrated in biological processes (BP), namely inflammatory response, response to cytokines, negative regulation of apoptotic signaling pathway, leukocyte migration and positive regulation of cell migration, leukocyte cell-cell adhesion, regulation of neurogenesis, negative regulation of intrinsic apoptotic signaling pathway and morphogenesis of epithelium, as well as one Kyoto Encyclopedia of Genes and Genomes (KEGG) that is leukocyte transendothelial migration (Figures 5A–C; Table 4).

**TABLE 3:** Nine hub genes and their functions.

Gene symbol	Description	Function
CXCL8	Chemokine (C-X-C motif) Ligand 8	attracts neutrophils, basophils, and T-cells, involving in neutrophil activation
ICAM1	Intercellular Adhesion Molecule 1	Encodes a cell surface glycoprotein which is typically expressed on endothelial cells and cells of the immune system.
CXCR4	Chemokine (C-X-C motif) Receptor 4	A role in regulation of cell migration
CD44	CD44 Molecule	lymphocyte activation, recirculation and homing, hematopoiesis, and tumor metastasis
IL1B	Interleukin 1 Beta	Potent proinflammatory cytokine
MMP9	Matrix Metallopeptidase 9	Involving in the breakdown of extracellular matrix in normal physiological processes and disease processes.
SPP1	Secreted Phosphoprotein 1	A cytokine involved in enhancing production of interferon-gamma and IL-12 and reducing production of IL-10 and is essential in the pathway that leads to type I immunity.
TIMP1	TIMP Metallopeptidase Inhibitor 1	Metalloproteinase inhibitor forming one to one complexes with target metalloproteinases, and irreversibly inactivates them by binding to their catalytic zinc cofactor.
HIF1A	Hypoxia Inducible Factor 1 Subunit Alpha	A master transcriptional regulator of the adaptive response to hypoxia. Plays an essential role in embryonic vascularization, tumor angiogenesis and pathophysiology of ischemic disease.

**TABLE 4:** Functional enrichment analysis of hub genes.

Term	Description	Count in gene set	-LogP	Gene symbol
GO:0006954	inflammatory response	7	10.91830694	CD44,HIF1A,IL1B,CXCL8,SPP1,TIMP1,CXCR4
GO:0034097	response to cytokine	7	9.518326992	CD44,HIF1A,ICAM1,IL1B,CXCL8,TIMP1,CXCR4
GO:2001234	negative regulation of apoptotic signaling pathway	5	8.550111382	CD44,HIF1A,ICAM1,IL1B,MMP9,
GO:0050900	positive regulation of angiogenesis	5	8.502623951	ICAM1,IL1B,CXCL8,MMP9,CXCR4
GO:0030335	positive regulation of cell migration	6	8.499677873	HIF1A,ICAM1,IL1B,CXCL8,MMP9,CXCR4
GO:0007159	leukocyte cell-cell adhesion	3	6.322506413	CD44,ICAM1,IL1B
GO:0050767	regulation of neurogenesis	4	5.618029472	HIF1A,IL1B,SPP1,CXCR4
GO:2001243	negative regulation of intrinsic apoptotic signaling pathway	3	5.50966188	CD44,HIF1A,MMP9
hsa04670	Leukocyte transendothelial migration	3	5.364159855	ICAM1,MMP9,CXCR4
GO:0002009	morphogenesis of an epithelium	3	3.60847974	CD44,HIF1A,CXCR4

### Establishment of miRNAs-Hub Genes Regulatory Network

**FIGURE 6:**
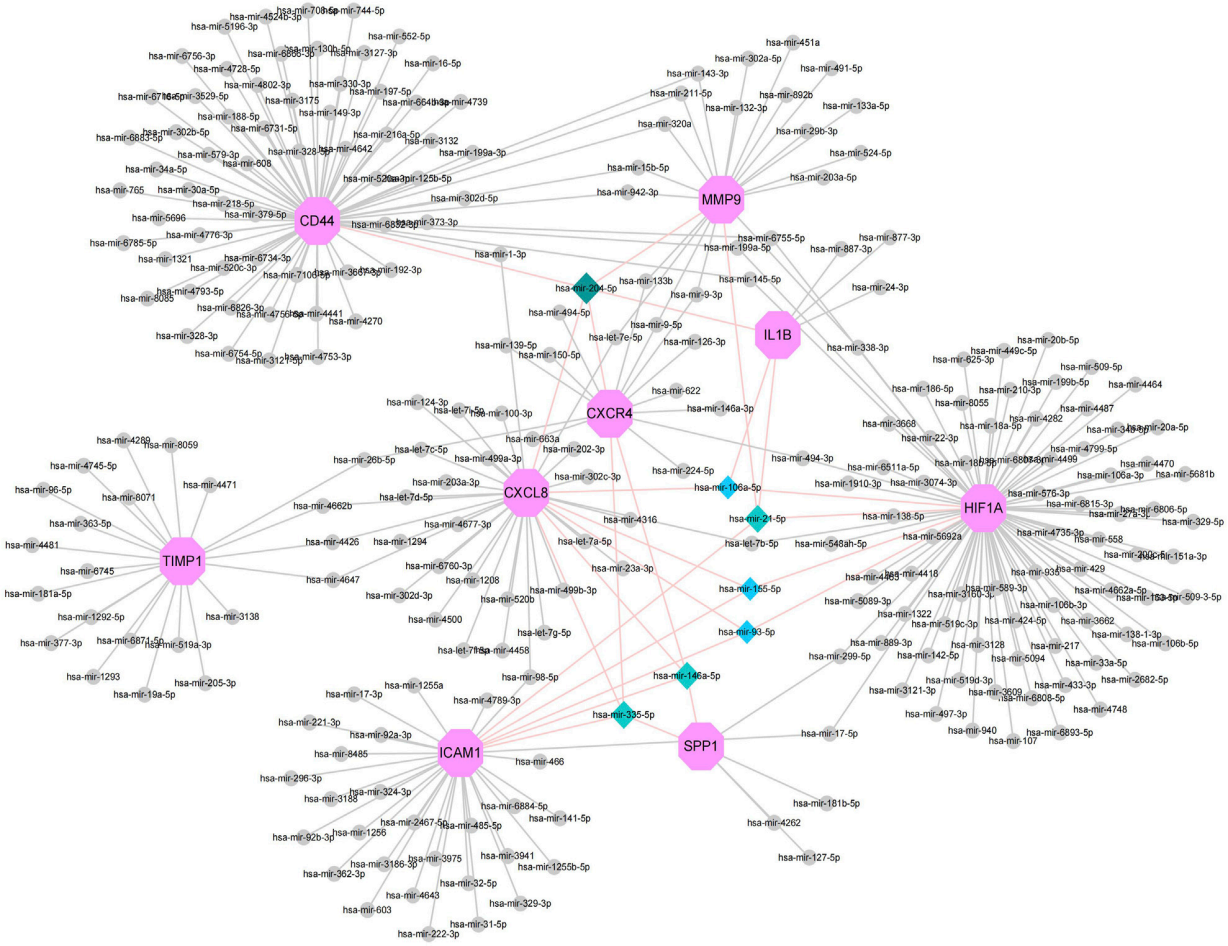
Top 9 hub genes in the integrated miRNA-DEGs network. The pink octagonal shape indicates the 9 hub genes. The gray circles indicate miRNAs with low connective properties to the hub genes. Dark green-light green-light blue diamonds indicate miRNAs with high connective properties to the hub genes.

MiRNAs play a variety of roles in the regulation of gene expression. Based on the Network Analyst database, Cytoscape was used to construct miRNAs-hub genes regulatory networks to identify miRNAs aimed at hub genes. Nine hub genes and their correspondent regulatory miRNAs molecules are shown in the Figure 6 and Supplementary Table S5. One miRNA (hsa-mir-204-5p) had 5 target genes (CD44, IL1B, MMP9, CXCR4 and CXCL8). Among the 9 hub genes, CXCL8, CXCR4, SPP1, and ICAM1 were common targets of 2 miRNAs (has-miR-335-5p and has-miR-146a-5p), while CXCL8, HIF1A, SPP1 and ICAM1 were common targets of 2 miRNAs (has-miR-155-5p and has-miR-93-5p).

## Discussion

Studies of genome-wide association have transformed the complex field of polygenic diseases and helped find several genes responsible for UC, resulting in new understanding of the pathogenesis of the disease.

The underlying causes of UC are ambiguous and complex, and its pathogenesis is multifactorial, with environmental and genetic factors interacting to produce a range of clinical manifestations. In this study, 446 DEGs were identified, consisting of 309 upregulated genes and 137 downregulated genes. The results of GO functional classification indicated that the DEGs were mainly enriched in extracellular matrix, regulation of cell adhesion, inflammatory response, response to cytokines, monocarboxylic acid metabolic process and response to toxic substances. KEGG pathway analysis indicated that the up-regulated DEGs were significantly enriched in Complement and coagulation cascades, Amoebiasis and TNF signaling pathway. In the PPI network of DEGs, 9 (CXCL8, ICAM1, CXCR4, CD44, IL1B, MMP9, SPP1, TIMP1, and HIF1A) out of 446 genes had high degree of interation. All of the 9 hub genes were up-regulated in patients with UC. GO analysis showed that these 9 genes were highly enriched in inflammatory response, response to cytokines, negative regulation of apoptotic signaling pathway, leukocyte migration and positive regulation of cell migration, leukocyte cell-cell adhesion, regulation of neurogenesis, negative regulation of intrinsic apoptotic signaling pathway and morphogenesis of epithelium, as well as KEGG that is leukocyte transendothelial migration. Triggers of IBD include chronic inflammatory responses triggered by specific pathogens or viral infections or mucosal barrier defects (Hanauer, 2006). Leukocyte migration is involved in the progression of IBD, so blocking leukocyte migration to the intestine is the major strategy used to control the disease and alleviate symptoms (Veny et al., 2020). It has been shown that the TNF signaling pathway is involved in the progression of UC(Danese et al., 2020). The results of these enriched GO terms and the KEGG pathway suggest that the DEGs or hub genes identified in this study may be involved in the progression of UC through the above-mentioned ways.CXCL8 (Chemokine (C-X-C motif) Ligand 8, IL-8), a classic ELR+CXC chemokine (Baird et al., 2011; Russo et al., 2014; Ha et al., 2017), is one of the first and most extensively studied chemokines that can be released by a wide range of cell types, including neutrophils (Bie et al., 2019), monocytes (Schroder et al., 1988), and macrophages (Wanninger et al., 2009). One of the most important pro-inflammatory factors is CXCL8, which acts as a key factor in numerous inflammatory diseases, including UC. Signaling pathways such as PI3K/Akt, MAPKs, and NF-*B are involved in the pathogenesis of UC through the CXCL8-CXCR1/2 axis (Zhu et al., 2021). In our study, CXCL8 was upregulated in patients with UC, suggesting a potential role as a biological target to predict and guide UC treatment in the future.

ICAM1 (Intercellular Adhesion Molecule 1), a member of the immunoglobulin superfamily, is a highly glycosylated transmembrane protein involved in the migration and transport of leukocytes to sites of inflammation (Dustin et al., 1986). Leukocyte trafficking has been identified as a specific target for the treatment of IBD and is a multi-step procedure involving integrins on leukocytes and their immunoglobulin superfamily counterparts as ligands, such as ICAM-1 (Rivera-Nieves et al., 2008; Mosli et al., 2014). Alicaforsen, which treats pouchitis and left-sided UC by inhibiting ICAM-1 production, has been granted orphan drug designation and is available as an unlicensed drug under international regulations (Greuter and Rogler, 2017).

CXCR4 (Chemokine (C-X-C motif) Receptor 4) is a type of amino acid rhodopsin-like G protein-coupled receptor (GPCR) (Wu et al., 2010). The number of immature plasma cells specifically overexpressing CXCR4 is increased in the peripheral blood of UC patients (Hosomi et al., 2011). Mina T Kitazume et al. demonstrated that unique IgG plasma cells exacerbate mucosal inflammation through infiltration of CXCR4 into the inflamed mucosa and activation of “pathogenic” intestinal CD14 macrophages via IgG-ICFcγR signaling, thereby having a critical impact on UC pathogenesis (Uo et al., 2013). Lin et al. suggested that CXCR4 may affect the function of micro vascular endothelial cells (MVECs) during UC and further inhibits the protein C system (PCS) through the JNK MAPK-c-Jun pathway and serves as a potential therapeutic target for UC (Lin et al., 2017). In our study and previous studies, CXCR4 was upregulated in UC, indicating that it was a key target for the treatment of UC.

CD44 (CD44 molecule) is a non-kinase transmembrane glycoprotein which is overexpressed in multiple cell types, as well as in cancer stem cells, and with a known role in the development and progression of cancer (Naor et al., 2002). When CD44 combines with and triggers its primary ligand, hyaluronic acid (HA), it results in the activation of cell signaling pathways, induces cell proliferation, enhances cell survival, mediates cytoskeletal changes, and strengthens cell movement (Ponta et al., 2003). A recent study found that multi-bioresponsive silk fibroin-based nanoparticles targeted against CD44 with on-demand cytoplasmic drug release capacity could alleviate UC (Gou et al., 2019).

IL1B (Interleukin 1 beta, IL-1β) is a critical mediator in the inflammatory response and is not merely crucial for host response and defense against pathogens, but also aggravates damage during chronic disease and acute tissue damage (Lopez-Castejon and Brough, 2011). Earlier studies have shown higher concentrations of IL-1β in the intestinal mucosa of patients with UC (Nakase et al., 2022). In our study, the expression of IL1B was low in normal human intestinal tissues, suggesting that IL1B may be a biological target in UC.

MMP9 (Matrix Metallopeptidase 9) is a member of metalloproteinases (MMPs) that plays an important role not only in tumor invasion and migration (Herszenyi et al., 2007; Herszenyi et al., 2008), but also in inflammation and remodeling in UC (Stallmach et al., 2000; von Lampe et al., 2000). In our study, MMP9 was up-regulated UC tissues compared with normal human colon and rectum tissues, suggesting that MMP9 may be a potential marker of disease activity in patients with UC.

SPP1 (Secreted Phosphoprotein 1/Osteopontin/OPN) is a chemokine-like matrix phosphoglycoprotein that not only affects cell proliferation and invasion, but is also involved in the invasive and metastatic processes of many cancers, and is considered as a prognostic and diagnostic marker for several cancers (Shevde and Samant, 2014). Giannos P et al. concluded from the bioinformatic analysis that SPP1 may be a potential biomarker of genes and a predictor of IFX treatment resistance in the development of UC and CRC (Giannos et al., 2022). Chen F et al. suggested that SPP1/OPN plays an important role in the immune response and is also involved in the mucosal protection mechanism of IBD (Chen et al., 2013). The relationship between SPP1 and UC remains poorly understood and needs to be further explored.

TIMP1 (namely Tissue inhibitor of metalloproteinase 1), is one of the four members of the glycoprotein group (TIMP1-4), and translocation of extracellular matrix is its primary function. TIMP1 is involved in a variety of pathological processes, including wound healing and cancer metastasis (Gardner and Ghorpade, 2003). Huang et al. suggested that TIMP1 is consistently upregulated during the pathology of UC-associated CRC (ucaCRC) and could be a potential biomarker of prognostic deterioration in CRC (Huang et al., 2019). Moreover, plasma TIMP-1 levels reflect to some extent the colonic mucosal expression of TIMP-1 in UC patients and have the prospect of becoming a biomarker for clinical diagnosis of UC as well as for predicting its severity and guiding further treatment (Wang et al., 2009).

Hypoxia-inducible factor (HIF) mediates the major transcriptional response to hypoxia and is a key regulator under hypoxic stress (Semenza, 2003). Three distinct HIF isoforms exist in mammals, of which HIF1A (Hypoxia Inducible Factor 1 Subunit Alpha) is commonly expressed (Wang and Semenza, 1993). Vitamin D/VDR signaling suppresses UC by inhibiting the activation of HIF-1α in colonic epithelial cells (Xue et al., 2021). Qi Lv et al. have shown that the suppression of HIF-1α-mediated glycolysis by costunolide acts specifically in the differentiation of Th17 cells and consequent UC relief, and it also provides a novel prospective for the immunometabolic treatment of colitis (Lv et al., 2021).

A major role of microRNAs (miRNAs) is to regulate the expression of most human genes; they play a crucial role in the pathogenesis of autoimmune diseases, such as UC (Zhou et al., 2021). MiR-93 may be involved in the pathogenic process of IBD through its involvement in the regulation of autophagic activity (Chen et al., 2014). Omidbakhsh A et al. suggested that the difference in miR106a expression in the peripheral blood of UC and Crohn’s disease (CD) patients in active versus inactive phases suggests that miR106a may act as a potential biomarker for diagnosing and monitoring of disease activity (Omidbakhsh et al., 2018). LuX et al. reported that inhibition of miR-21-5p during UC rats reduced inflammation and apoptosis levels in RAW264.7 cells, suggesting a potential therapeutic role for miR-21-5p in human UC (Lu et al., 2020). Jabandziev et al. analyzed miRNA expression in the colonic tissue of 60 pediatric UC patients and identified miR-146a-5p as a diagnostic factor for primary sclerosing cholangitis in UC patients (Jabandziev et al., 2021). MiRNAs are involved in tumor formation in UC, and patients with UC have an increased risk of developing colitis-associated cancer (CAC) over 8 years (Chen et al., 2016). It has been reported that miR-155-5p promotes the metastasis of colon cancer cells by targeting the AU-rich element (ARE) in the 3′-UTR region of HuR mRNA to upregulate human antigen R (HuR); colon cancer cells could be inhibited from migrating when miR-155-5p is blocked from binding to the ARE in HuR (Al-Haidari et al., 2018). Our current findings suggest that several miRNAs, including miR-335-5p, mir-204-5p, miR-93-5p, miR106a-5p, miR-21-5p, miR-146a-5p, and miR-155-5p, may play key roles in UC. In addition, studies about genes and miRNAs in UC remains to be limited.

There is no doubt that the gene-miRNA regulatory network plays an important role in UC pathophysiology. This increases the understanding of UC and provides targeted therapeutic strategies and predictions for UC. One limitation of the study was that the microarray expression profiles were analyzed using bioinformatics analysis without being validated with basic experiments. In addition, we did not explore the detailed mechanisms of how the hub genes and miRNA regulate UC. Therefore, there is need to validate our findings using more clinical samples and further studies.

## Conclusion

In summary, we identified DEGs that can be potentially participating in the progression or occurrence of UC. A total of 446 DEGs,9 hub genes and 7 miRNAs were identified, which may be considered as biomarkers of UC. However, still further studies are necessary to clarify the biological functions of such genes in UC.

## Data Availability

Publicly available datasets were analyzed in this study. The names of the repository/repositories and accession number(s) can be found in the article/Supplementary Material.
