# Supplementary material for: Identification of Differentially Expressed Genes and miRNAs for Ulcerative Colitis Using Bioinformatics Analysis
Source: Front Genet. 2022 Jun 2;13:914384. doi: 10.3389/fgene.2022.914384 (PMC9201719; doi:10.3389/fgene.2022.914384)
Supplement: Supplementary file 1 [file Table1.docx]

Supplementary Table 1. GO terms for up-regulated DEGs between the control and UC.

| **Category** | **Term** | **Description** | **-LogP** | **InTerm_InList** |
| --- | --- | --- | --- | --- |
| GO Cellular Components | GO:0031012 | extracellular matrix | 37.41740768 | 56/568 |
| GO Biological Processes | GO:0030155 | regulation of cell adhesion | 31.69441117 | 57/760 |
| GO Biological Processes | GO:0006954 | inflammatory response | 30.19235198 | 47/504 |
| GO Biological Processes | GO:0034097 | response to cytokine | 27.73821405 | 54/799 |
| GO Biological Processes | GO:2000147 | positive regulation of cell motility | 26.36028272 | 46/584 |
| GO Biological Processes | GO:0001944 | vasculature development | 21.37895066 | 39/526 |
| GO Biological Processes | GO:0050778 | positive regulation of immune response | 21.36220647 | 40/559 |
| GO Biological Processes | GO:0045087 | innate immune response | 20.63339872 | 45/764 |
| GO Biological Processes | GO:0001819 | positive regulation of cytokine production | 18.17781181 | 34/475 |
| GO Biological Processes | GO:0010942 | positive regulation of cell death | 17.51927368 | 37/604 |
| GO Biological Processes | GO:0052547 | regulation of peptidase activity | 16.87295748 | 32/456 |
| GO Biological Processes | GO:0030198 | extracellular matrix organization | 16.21146196 | 25/266 |
| GO Biological Processes | GO:0001775 | cell activation | 14.36196498 | 34/636 |
| GO Biological Processes | GO:0002697 | regulation of immune effector process | 14.27577121 | 26/353 |
| GO Cellular Components | GO:0045121 | membrane raft | 14.14544716 | 25/326 |
| GO Biological Processes | GO:0098609 | cell-cell adhesion | 14.10398649 | 31/533 |
| GO Cellular Components | GO:0030667 | secretory granule membrane | 13.62778396 | 24/312 |
| GO Biological Processes | GO:0002683 | negative regulation of immune system process | 13.24574391 | 27/425 |
| GO Biological Processes | GO:0009611 | response to wounding | 13.12404485 | 27/430 |
| GO Biological Processes | GO:1901652 | response to peptide | 13.02790389 | 27/434 |
