# Supplementary material for: Identification of Differentially Expressed Genes and miRNAs for Ulcerative Colitis Using Bioinformatics Analysis
Source: Front Genet. 2022 Jun 2;13:914384. doi: 10.3389/fgene.2022.914384 (PMC9201719; doi:10.3389/fgene.2022.914384)
Supplement: Supplementary file 2 [file Table2.docx]

Supplementary Table 2. GO terms for down-regulated DEGs between the control and UC.

| **Category** | **Term** | **Description** | **-LogP** | **InTerm_InList** |
| --- | --- | --- | --- | --- |
| GO Biological Processes | GO:0032787 | monocarboxylic acid metabolic process | 10.94533055 | 18/492 |
| GO Biological Processes | GO:0009636 | response to toxic substance | 10.34241048 | 13/229 |
| GO Molecular Functions | GO:0016614 | oxidoreductase activity, acting on CH-OH group of donors | 9.096929766 | 10/138 |
| GO Molecular Functions | GO:0046943 | positive regulation of small molecule metabolic process | 7.185716917 | 9/164 |
| GO Cellular Components | GO:0045177 | carboxylic acid transmembrane transporter activity | 7.072029042 | 13/428 |
| GO Biological Processes | GO:0062013 | apical part of cell | 6.371838437 | 8/149 |
| GO Biological Processes | GO:1901615 | organic hydroxy compound metabolic process | 5.782472594 | 12/469 |
| GO Biological Processes | GO:0031667 | response to nutrient levels | 5.190880572 | 11/446 |
| GO Molecular Functions | GO:0004955 | prostaglandin receptor activity | 4.970246234 | 3/10 |
| GO Biological Processes | GO:0006730 | carbonated dehydratase activity | 4.530535071 | 4/39 |
| GO Biological Processes | GO:1901569 | fatty acid derivative catabolic process | 4.309910577 | 3/16 |
| GO Cellular Components | GO:0042622 | photoreceptor outer segment membrane | 4.309910577 | 3/16 |
| GO Biological Processes | GO:0070848 | nucleobase-containing small molecule metabolic process | 4.016012607 | 10/498 |
| GO Cellular Components | GO:0005903 | response to growth factor | 3.980768574 | 5/102 |
| GO Molecular Functions | GO:0015318 | anion transmembrane transport activity | 3.889505146 | 12/730 |
| GO Biological Processes | GO:0055086 | inorganic molecular entity transmembrane transporter activity | 3.756834341 | 10/536 |
| GO Molecular Functions | GO:0016829 | lyase activity | 3.538957621 | 6/197 |
| GO Biological Processes | GO:0051223 | monovalent inorganic cation homeostasis | 3.349790742 | 9/495 |
| GO Biological Processes | GO:0030001 | regulation of protein transport | 3.285834486 | 10/615 |
| GO Biological Processes | GO:0009225 | metal ion transport | 3.269578854 | 3/35 |
