# Supplementary material for: Identification of Differentially Expressed Genes and miRNAs for Ulcerative Colitis Using Bioinformatics Analysis
Source: Front Genet. 2022 Jun 2;13:914384. doi: 10.3389/fgene.2022.914384 (PMC9201719; doi:10.3389/fgene.2022.914384)
Supplement: Supplementary file 3 [file Table5.docx]

Supplementary Table 5. The critical miRNAs in UC.

| **Name** | **Degree** | **Genes of Interaction** | **Betweenness Score** |
| --- | --- | --- | --- |
| hsa-mir-204-5p | 5 | CD44, MMP9, IL1B, CXCR4, CXCL8 | 4149.65 |
| hsa-mir-21-5p | 4 | MMP9, IL1B, HIF1A, ICAM1 | 2506.85 |
| hsa-mir-146a-5p | 4 | CXCL8, CXCR4, ICAM1, SPP1 | 1097.6 |
| hsa-mir-335-5p | 4 | CXCL8, CXCR4, ICAM1, SPP1 | 1097.6 |
| hsa-mir-93-5p | 3 | CXCL8, HIFA1, ICAM1 | 2041.6 |
| hsa-mir-155-5p | 3 | CXCL8, HIFA1, ICAM1 | 2041.6 |
| hsa-mir-106a-5p | 3 | CXCL8, HIFA1, IL1B | 1183.03 |
