# Supplementary material for: Identification of Differentially Expressed Genes and miRNAs for Ulcerative Colitis Using Bioinformatics Analysis
Source: Front Genet. 2022 Jun 2;13:914384. doi: 10.3389/fgene.2022.914384 (PMC9201719; doi:10.3389/fgene.2022.914384)
Supplement: Supplementary file 4 [file Table3.docx]

Supplementary Table 3. KEGG pathways for up-regulated DEGs between the control and UC.

| **Category** | **Term** | **Description** | **-LogP** | **InTerm_InList** |
| --- | --- | --- | --- | --- |
| KEGG Pathway | hsa04610 | Complement and coagulation cascades | 15.38930231 | 16/85 |
| KEGG Pathway | hsa04668 | TNF signaling pathway | 12.18925026 | 15/112 |
| KEGG Pathway | hsa05146 | Amoebiasis | 11.56459875 | 14/102 |
| KEGG Pathway | hsa05150 | Staphylococcus aureus infection | 10.70377702 | 13/96 |
| KEGG Pathway | hsa04151 | PI3K-Akt signaling pathway | 8.256337386 | 19/354 |
| KEGG Pathway | hsa05144 | Malaria | 7.391646355 | 8/50 |
| KEGG Pathway | hsa04621 | NOD-like receptor signaling pathway | 7.198532035 | 13/184 |
| KEGG Pathway | hsa05200 | Pathways in cancer | 6.776625028 | 21/531 |
| KEGG Pathway | hsa04670 | Leukocyte transendothelial migration | 6.53806355 | 10/114 |
| KEGG Pathway | hsa04613 | Neutrophil extracellular trap formation | 6.166048837 | 12/190 |
| KEGG Pathway | hsa04380 | Osteoclast differentiation | 6.06989502 | 10/128 |
| KEGG Pathway | hsa04926 | Relaxin signaling pathway | 6.038756236 | 10/129 |
| KEGG Pathway | hsa05205 | Proteoglycans in cancer | 5.817653638 | 12/205 |
| KEGG Pathway | hsa04662 | B cell receptor signaling pathway | 5.692656885 | 8/82 |
| KEGG Pathway | hsa05340 | Primary immunodeficiency | 5.639868976 | 6/38 |
| KEGG Pathway | hsa05202 | Transcriptional misregulation in cancer | 5.269695607 | 11/193 |
| KEGG Pathway | hsa04640 | Hematopoietic cell lineage | 5.076345051 | 8/99 |
| KEGG Pathway | hsa04620 | Toll-like receptor signaling pathway | 4.918113228 | 8/104 |
| KEGG Pathway | hsa05166 | Human T-cell leukemia virus 1 infection | 3.977056761 | 10/222 |
| KEGG Pathway | hsa05222 | Small cell lung cancer | 3.428434514 | 6/92 |
