# Supplementary material for: Identification of Differentially Expressed Genes and miRNAs for Ulcerative Colitis Using Bioinformatics Analysis
Source: Front Genet. 2022 Jun 2;13:914384. doi: 10.3389/fgene.2022.914384 (PMC9201719; doi:10.3389/fgene.2022.914384)
Supplement: Supplementary file 5 [file Table4.docx]

Supplementary Table 4. KEGG pathways for down-regulated DEGs between the control and UC.

| **Category** | **Term** | **Description** | **-LogP** | **InTerm_InList** |
| --- | --- | --- | --- | --- |
| KEGG Pathway | hsa04976 | Bile secretion | 9.53494011 | 9/89 |
| KEGG Pathway | hsa04978 | Mineral absorption | 6.495271081 | 6/60 |
| KEGG Pathway | hsa00071 | Fatty acid degradation | 5.82490095 | 5/43 |
| KEGG Pathway | hsa00910 | Nitrogen metabolism | 4.227033872 | 3/17 |
| KEGG Pathway | hsa00620 | Pyruvate metabolism | 4.206573622 | 4/47 |
| KEGG Pathway | hsa04964 | Proximal tubule bicarbonate reclamation | 3.819983251 | 3/23 |
| KEGG Pathway | hsa00590 | Arachidonic acid metabolism | 3.761665958 | 4/61 |
| KEGG Pathway | hsa00140 | Steroid hormone biosynthesis | 3.761665958 | 4/61 |
| KEGG Pathway | hsa03320 | PPAR signaling pathway | 3.41577296 | 4/75 |
| KEGG Pathway | hsa00040 | Pentose and glucuronate interconversions | 3.307057968 | 3/34 |
| KEGG Pathway | hsa04146 | Peroxisome | 3.268337935 | 4/82 |
| KEGG Pathway | hsa00480 | Glutathione metabolism | 2.629163274 | 3/58 |
| KEGG Pathway | hsa04151 | PI3K-Akt signaling pathway | 2.246913147 | 6/354 |
